# Development and validation of a trans-ancestry polygenic risk score for type 2 diabetes in diverse populations

**DOI:** 10.1186/s13073-022-01074-2

**Published:** 2022-06-29

**Authors:** Tian Ge, Marguerite R. Irvin, Amit Patki, Vinodh Srinivasasainagendra, Yen-Feng Lin, Hemant K. Tiwari, Nicole D. Armstrong, Barbara Benoit, Chia-Yen Chen, Karmel W. Choi, James J. Cimino, Brittney H. Davis, Ozan Dikilitas, Bethany Etheridge, Yen-Chen Anne Feng, Vivian Gainer, Hailiang Huang, Gail P. Jarvik, Christopher Kachulis, Eimear E. Kenny, Atlas Khan, Krzysztof Kiryluk, Leah Kottyan, Iftikhar J. Kullo, Christoph Lange, Niall Lennon, Aaron Leong, Edyta Malolepsza, Ayme D. Miles, Shawn Murphy, Bahram Namjou, Renuka Narayan, Mark J. O’Connor, Jennifer A. Pacheco, Emma Perez, Laura J. Rasmussen-Torvik, Elisabeth A. Rosenthal, Daniel Schaid, Maria Stamou, Miriam S. Udler, Wei-Qi Wei, Scott T. Weiss, Maggie C. Y. Ng, Jordan W. Smoller, Matthew S. Lebo, James B. Meigs, Nita A. Limdi, Elizabeth W. Karlson

**Affiliations:** 1 Center for Genomic Medicine, Massachusetts General Hospital, Boston, MA USA; 2 Center for Precision Psychiatry, Massachusetts General Hospital, Boston, MA USA; 3 Department of Psychiatry, Massachusetts General Hospital, Boston, MA USA; 4 Broad Institute of MIT and Harvard, Cambridge, MA USA; 5 Department of Epidemiology, School of Public Health, University of Alabama at Birmingham, Birmingham, AL USA; 6 Department of Biostatistics, School of Public Health, University of Alabama at Birmingham, Birmingham, AL USA; 7 Center for Neuropsychiatric Research, National Health Research Institutes, Miaoli, Taiwan; 8 Department of Public Health & Medical Humanities, School of Medicine, National Yang Ming Chiao Tung University, Taipei, Taiwan; 9 Institute of Behavioral Medicine, College of Medicine, National Cheng Kung University, Tainan, Taiwan; 10 Mass General Brigham Research Information Science & Computing, Boston, MA USA; 11 Translational Biology, Biogen Inc., Cambridge, MA USA; 12 Informatics Institute, University of Alabama at Birmingham, Birmingham, AL USA; 13 Department of Neurology, School of Medicine, University of Alabama at Birmingham, Birmingham, AL USA; 14 Department of Cardiovascular Medicine, Mayo Clinic, Rochester, MN USA; 15 Department of Internal Medicine, Mayo Clinician-Investigator Training Program, Mayo Clinic, Rochester, MN USA; 16 Institute of Epidemiology and Preventive Medicine, National Taiwan University, Taipei, Taiwan; 17 Department of Medicine, Massachusetts General Hospital, Boston, MA USA; 18 Analytic and Translational Genetics Unit, Massachusetts General Hospital, Boston, MA USA; 19 Division of Medical Genetics, Department of Medicine, University of Washington, Seattle, WA USA; 20 Institute for Genomic Health, Icahn School of Medicine at Mount Sinai, New York, NY USA; 21 Division of Nephrology, Department of Medicine, Vagelos College of Physicians & Surgeons, Columbia University, New York, USA; 22 Center for Autoimmune Genomics and Etiology, Cincinnati Children’s Hospital Medical Center, Cincinnati, OH USA; 23 Department of Biostatistics, Harvard T.H. Chan School of Public Health, Boston, MA USA; 24 Division of General Internal Medicine, Massachusetts General Hospital, Boston, MA USA; 25 Diabetes Unit, Massachusetts General Hospital, Boston, MA USA; 26 Department of Neurology, Massachusetts General Hospital, Boston, MA USA; 27 UMass Memorial Health Care, Worcester, MA USA; 28 Center for Genetic Medicine, Feinberg School of Medicine, Northwestern University, Chicago, IL USA; 29 Department of Medicine, Brigham and Women’s Hospital, Boston, MA USA; 30 Mass General Brigham Personalized Medicine, Boston, MA USA; 31 Department of Preventive Medicine, Feinberg School of Medicine, Northwestern University, Chicago, IL USA; 32 Department of Quantitative Health Sciences, Mayo Clinic, Rochester, MN USA; 33 Division of Endocrinology, Massachusetts General Hospital, Boston, MA USA; 34 Department of Biomedical Informatics, Vanderbilt University Medical Center, Nashville, TN USA; 35 Channing Division of Network Medicine, Department of Medicine, Brigham and Women’s Hospital, Boston, MA USA; 36 Vanderbilt Genetics Institute, Division of Genetic Medicine, Vanderbilt University Medical Center, Nashville, TN USA; 37 Department of Pathology, Brigham and Women’s Hospital, Boston, MA USA

**Keywords:** Polygenic risk score, Type 2 diabetes, Diverse populations, Clinical implementation

## Abstract

**Background:**

Type 2 diabetes (T2D) is a worldwide scourge caused by both genetic and environmental risk factors that disproportionately afflicts communities of color. Leveraging existing large-scale genome-wide association studies (GWAS), polygenic risk scores (PRS) have shown promise to complement established clinical risk factors and intervention paradigms, and improve early diagnosis and prevention of T2D. However, to date, T2D PRS have been most widely developed and validated in individuals of European descent. Comprehensive assessment of T2D PRS in non-European populations is critical for equitable deployment of PRS to clinical practice that benefits global populations.

**Methods:**

We integrated T2D GWAS in European, African, and East Asian populations to construct a trans-ancestry T2D PRS using a newly developed Bayesian polygenic modeling method, and assessed the prediction accuracy of the PRS in the multi-ethnic Electronic Medical Records and Genomics (eMERGE) study (11,945 cases; 57,694 controls), four Black cohorts (5137 cases; 9657 controls), and the Taiwan Biobank (4570 cases; 84,996 controls). We additionally evaluated a post hoc ancestry adjustment method that can express the polygenic risk on the same scale across ancestrally diverse individuals and facilitate the clinical implementation of the PRS in prospective cohorts.

**Results:**

The trans-ancestry PRS was significantly associated with T2D status across the ancestral groups examined. The top 2% of the PRS distribution can identify individuals with an approximately 2.5–4.5-fold of increase in T2D risk, which corresponds to the increased risk of T2D for first-degree relatives. The post hoc ancestry adjustment method eliminated major distributional differences in the PRS across ancestries without compromising its predictive performance.

**Conclusions:**

By integrating T2D GWAS from multiple populations, we developed and validated a trans-ancestry PRS, and demonstrated its potential as a meaningful index of risk among diverse patients in clinical settings. Our efforts represent the first step towards the implementation of the T2D PRS into routine healthcare.

**Supplementary Information:**

The online version contains supplementary material available at 10.1186/s13073-022-01074-2.

## Background

Type 2 diabetes (T2D) is a common, chronic disease caused by both genetic and environmental risk factors and their interactions [1], which has significantly increased prevalence in the past 20 years [2] and disproportionately afflicts communities of color [3–5]. The current screening of T2D focuses on individuals with demographic and clinical risk factors, including overweight or obesity, age >35 years, and a family history of diabetes [6]. However, despite preventative strategies and public health efforts to improve nutrition and physical activity, facilitate access to care, and limit tobacco and alcohol use, the morbidity and mortality associated with T2D remain unaltered [5], likely because most interventions are adopted too late in the course of disease trajectory.

Recent large-scale genome-wide association studies (GWAS) in diverse populations have identified hundreds of genetic loci associated with T2D [7–9]. Polygenic risk scores (PRS), which aggregate the genetic risk of individual alleles across the genome, are thus promising to predict future T2D occurrence and improve early diagnosis, intervention, and prevention of T2D [10–15]. However, to date, T2D PRS were most widely developed and validated in individuals of European descent. Given that the predictive performance of PRS often attenuates in non-European populations [16], and communities of color are experiencing continuing increased rates of T2D [2–5], it is critically important to assess and optimize the transferability of T2D PRS in diverse populations before they can be deployed in clinical settings.

The Electronic Medical Records and Genomics (eMERGE) study is a consortium of US medical research institutions with a goal to develop and disseminate methods and best practices for utilization of electronic medical records (EMR) in genomic research. Phase IV of the eMERGE study aims to establish protocols and methodologies for improved genetic risk assessment, and integrate genomic signatures, including monogenic and polygenic risk, and clinical risk factors into routine medical practice to identify individuals at high disease risk and recommend intervention strategies [17]. Towards this end, the consortium has identified 10 diseases, including T2D, where the predictive power of PRS, combined with clinical risk factors and intervention/treatment paradigms, has shown promise to delay, mitigate, prevent, or manage the disease. Here, as part of the eMERGE IV study, we present the construction and evaluation of a trans-ancestry T2D PRS to address the opportunities and challenges in the clinical translation of T2D PRS. Specifically, we integrated T2D GWAS in European, African, and East Asian individuals to construct the trans-ancestry PRS using state-of-the-art Bayesian polygenic modeling methods [18], and evaluated the PRS in the multi-ethnic eMERGE I-III samples [19], four Black cohorts, and the Taiwan Biobank [20, 21]. We additionally assessed a post hoc ancestry adjustment method that can express the polygenic risk on the same scale across populations and facilitate the use of a single cutoff to identify high-risk individuals with ancestrally diverse backgrounds in prospective cohorts. Our efforts represent the first step towards the implementation of T2D PRS into routine clinical care.

## Methods

### Construction of trans-ancestry T2D PRS from published T2D GWAS

We identified three large-scale T2D GWAS conducted in different populations to derive a trans-ancestry T2D PRS: (i) a GWAS in individuals of European descent with 74,124 T2D cases and 824,006 controls [8]; (ii) a meta-GWAS of T2D in African Americans performed by the MEta-analysis of type 2 Diabetes in African Americans (MEDIA) Consortium with 8,284 cases and 15,543 controls [22]; and (iii) a GWAS in the Japanese population with 45,383 cases and 132,032 controls, performed by Biobank Japan (BBJ) [23].

To empirically examine the concordance of genetic effects on T2D across different populations, we extracted the lead variant of each genome-wide significant locus in the fixed-effect meta-analysis of the three GWAS and compared their per-allele effect sizes across populations. When the lead variant was missing in a population, we used the tag variant that was most strongly correlated with the lead variant with an *R*^2^ no smaller than 0.6. We note that the MEDIA T2D GWAS was imputed to HapMap reference panels and included a relatively small number of genetic variants (~2.5M) across the genome. As a result, the large majority of the variants representing the African population were tag variants which may lead to under-estimation of the effect size concordance between ancestries.

We used PRS-CSx, a recently developed Bayesian polygenic modeling method, to construct the trans-ancestry PRS [18]. PRS-CSx jointly models the three GWAS summary statistics and couples genetic effects across populations using a shared continuous shrinkage prior, which enables more accurate effect size estimation by sharing information between summary statistics and leveraging linkage disequilibrium (LD) diversity across discovery samples. The shared prior allows for correlated but varying effect size estimates across populations, retaining the flexibility of the modeling framework. In addition, PRS-CSx accounts for population-specific allele frequencies and LD patterns and inherits efficient and robust posterior inference algorithms (Gibbs sampler) from PRS-CS [24]. We used pre-computed 1000 Genomes Project (1KG) [25] reference panels that matched the ancestry of each discovery GWAS, and a fully Bayesian algorithm for model fitting, which automatically learned all model parameters from the summary statistics without the need for hyper-parameter tuning. Population-specific posterior effect size estimates were combined using an inverse-variance-weighted meta-analysis within the Gibbs sampler (via the “--meta” option provided by the software). The final PRS-CSx output included 1,259,754 HapMap3 variants and their weights, which can be applied to any genotyped individual not included in the discovery GWAS to calculate a polygenic risk score.

### Overview of the evaluation of the trans-ancestry PRS

**Fig. 1:**
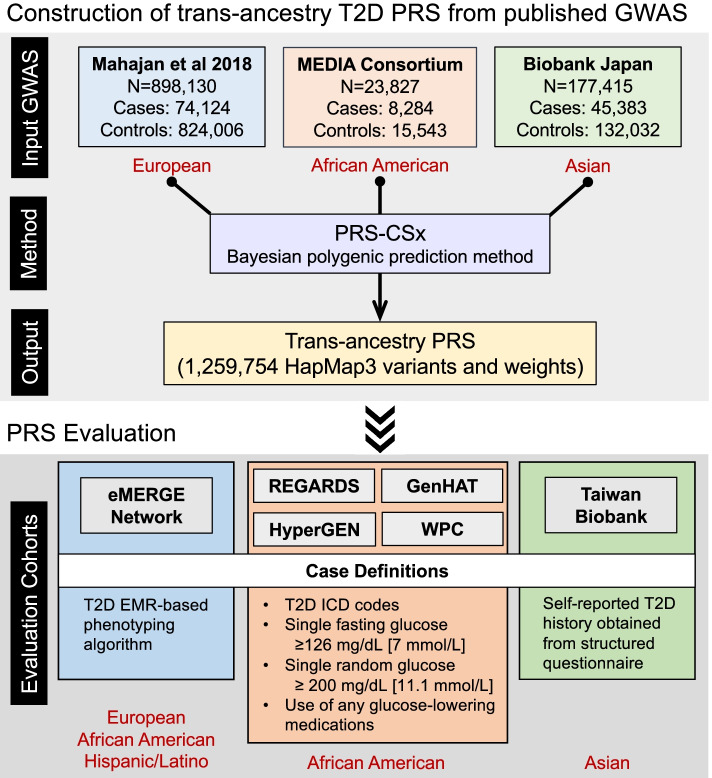
Workflow of the construction and evaluation of the trans-ancestry T2D PRS

We first evaluated the trans-ancestry PRS constructed by PRS-CSx among the European, African and Hispanic/Latino individuals in the eMERGE study [17, 19, 26]. To define T2D cases and controls in eMERGE, we validated an EMR-based phenotyping algorithm of T2D to apply across the eMERGE samples (see below). We benchmarked the prediction accuracy of the PRS-CSx-derived trans-ancestry PRS against three alternative scores: (i) a European-specific score derived by applying PRS-CS-auto [24] to the European T2D GWAS summary statistics (PRS-CS EUR); (ii) a trans-ancestry score derived by applying PRS-CS-auto to the meta-analysis of the European [8], MEDIA [22], and BBJ [23] T2D GWAS (PRS-CS Meta); (iii) a trans-ancestry score derived by applying LDpred2-auto [27] to the T2D meta-GWAS (LDpred2 Meta). Given that individuals of African descent were underrepresented in the discovery GWAS, the predictive performance of the PRS was expected to be lower in African individuals. We next evaluated the trans-ancestry PRS in four independent self-reported Black cohorts—REGARDS [28], GenHAT [29], HyperGEN [30], and WPC [31]—collected by the University of Alabama at Birmingham (UAB). In all four UAB cohorts, T2D cases were defined with T2D ICD codes, a single measurement of glucose (fasting glucose ≥126 mg/dL [7 mmol/L] or random glucose ≥ 200 mg/dL [11.1 mmol/L]) or use of any glucose-lowering medications. Lastly, since the number of Asian participants in eMERGE was low, precluding the evaluation of the PRS in the Asian population, we sought to assess the trans-ancestry PRS in the Taiwan Biobank (TWB), a community-based prospective cohort study of the Taiwanese population, aged 30 to 70 years old at recruitment [20, 21]. TWB participants were interviewed using a structured questionnaire at one of the recruitment centers, which included questions on basic demographic information, lifestyle, environmental exposures, reproductive history, medical history, and family history. Participants with self-reported T2D history were defined as cases in the PRS analysis. After removing 1,776 REGARDS samples that overlapped with the MEDIA study, there was no remaining overlap between the discovery GWAS samples and participants in the evaluation cohorts. Figure 1 summarizes the construction and evaluation of the trans-ancestry T2D PRS derived by PRS-CSx in this work. Below, we briefly describe the phenotyping algorithm in eMERGE and the sample characteristics and processing of genetic data in each evaluation dataset.

### Validation of EMR-based phenotyping algorithms to classify T2D in eMERGE

We adapted two prior phenotyping algorithms, one from eMERGE developed in 2011 [32] and one from Mass General Brigham (MGB) developed in 2014 [33]. The eMERGE algorithm was a rule-based algorithm originally developed at Northwestern University. Three eMERGE sites conducted chart reviews of cases and controls defined by the algorithm with sample sizes ranging from 50 to 100 cases and 44 to 50 controls, demonstrating 98% positive predictive value (PPV) for cases and 100% negative predictive value (NPV) for controls. The eMERGE algorithm was modified to remove the exclusion of charts with at least one ICD9 code for type 1 diabetes to improve sensitivity, to add ICD10 codes, and to add new diabetes medications released since 2011 (referred to as the “modified eMERGE algorithm”). The MGB algorithm was a machine learning-based algorithm using the PheCAP method [33] that had 90% PPV among a chart review dataset that was screen positive for one of 19 phenotypes (prevalence of definite or possible T2D 16%). This algorithm was modified to add ICD10 codes and to add new diabetes medications released since 2014 (referred to as the “modified MGB algorithm”).

We tested the performance of the original eMERGE algorithm, modified eMERGE algorithm, and modified MGB algorithm against two independent gold standard datasets derived from chart reviews, one set from MGB EMR, and one set from the UAB EMR. We selected charts for review by applying a data floor of at least one clinical note and at least three ICD9/10 codes from distinct dates, and screened for at least one ICD9/10 code for T2D. We conducted chart reviews for 208 randomly selected screen positive subjects from MGB and 198 screen positive subjects from UAB. In addition, we conducted chart reviews for 200 screen negative subjects from UAB. At MGB, charts were reviewed for diagnostic criteria for T2D by two endocrinology fellows, initially with 20 charts reviewed by both reviewers, to assess concordance. A chart review guideline was developed and used for reviewing the remaining charts, divided into two independent sets. At UAB, charts were reviewed by a trained study coordinator using the same chart review guideline.

### eMERGE genetic data

We used genetic data from 8 eMERGE sites in this work: Cincinnati Children's Hospital Medical Center (CCHMC), Children’s Hospital of Pennsylvania (CHOP), Columbia University, Mass General Brigham (MGB), Mayo Clinic, Icahn School of Medicine at Mount Sinai, Northwestern University (NU), and Vanderbilt University Medical Center (VUMC). Imputed genome-wide data against the Haplotype Reference Consortium (HRC) across the 8 sites were obtained from the eMERGE Network [17, 19]. We merged all eMERGE samples with the 1KG phase 3 data (*N*=2504), and selected high-quality, common variants shared between the two datasets. We pruned the merged dataset (PLINK command --indep-pairwise 500 50 0.05), retaining a set of independent variants, and calculated principal components (PCs) in the 1KG samples using the LD-pruned variants. We then projected eMERGE samples into the 1KG PC space and grouped each eMERGE sample with one of the four 1KG super-populations—European [EUR], African [AFR], Admixed American [AMR], and East Asian [EAS]—by co-clustering the projected eMERGE samples with the 1KG reference samples. Continental ancestry memberships were verified by visual inspection of the PC plots (Additional File 1: Fig. S1). We further intersected genetically inferred ancestry with self-reported race/ethnicity, namely White and non-Hispanic/Latino, Black or African American, Hispanic or Latino, and Asian, for the four ancestral groups, respectively, and randomly removed one sample from each pair of related individuals (kingship coefficient >0.1), leaving 54,793 European, 12,472 African, 2,374 Hispanic/Latino and 557 East Asian individuals with T2D case and control definitions (Table 1; Additional File 2: Table S1). We did not use Asian samples in subsequent PRS analyses due to the small sample size. Variants with minor allele frequency (MAF) <1% within each population were excluded.

**Table 1:** Sample characteristics of the evaluation datasets

		Age (Mean ± SD)	Sex (Female %)	*N* case	*N* control
eMERGE	European	59.4 ± 23.2	51.3%	8389	46,404
	African	45.8 ± 22.9	60.0%	2688	9784
	Hispanic/Latino	56.3 ± 20.6	60.5%	868	1506
UAB Black Cohorts	REGARDS	63.8 ± 9.3	60.5%	1659	5086
	GenHAT	66.1 ± 7.5	55.3%	2776	2722
	HyperGEN	47.0 ± 12.8	63.5%	402	1494
	WPC	57.5 ± 15.2	57.6%	300	355
Taiwan Biobank	Batch 1	48.9 ± 11.1	49.3%	1248	23,862
	Batch 2	50.5 ± 10.5	68.6%	2806	51,272
	Batch 3	49.3 ± 10.9	65.7%	516	9862

### Reasons for Geographic and Racial Differences in Stroke Study (REGARDS)

REGARDS [28] is a national, population-based, longitudinal study of incident stroke and associated risk factors of over 30,000 self-reported Black and White adults aged 45 years or older from all 48 contiguous U.S. states and the District of Columbia. REGARDS was designed to investigate reasons underlying the higher rate of stroke mortality among Black compared to White individuals, as well as why residents of the Southeastern U.S. had worse death rates compared to other US regions. By design, participants were oversampled if they were residents of the stroke belt or if they were Black. Participants completed a computer-assisted telephone interview to collect demographic information and medication adherence, and an in-home visit for blood pressure measurements and collection of blood and urine samples. Participants have been contacted on 6-month intervals to obtain information on incident stroke or secondary outcomes. Genotyping was performed on 8,916 Black participants using Illumina MEGA arrays. Imputation was conducted using release 2 (r2) of the National Heart Lung and Blood Institute (NHLBI) TOPMed reference panel available through the BioData Catalyst framework. Participants were excluded if they had sex mismatch or genotyping call rate <0.95, or if they were duplicates or an outlier in the principal component analysis (PCA; outside of 6 standard deviations), resulting in 6,745 individuals (Table 1). Imputed variants were inspected for their imputation quality scores (*R*^2^) and it was noted that more than 99% of the variants with MAF >1% had an imputation quality >0.6. Given the high quality in the imputed callset for variants with MAF >1%, genotypes with genotypic probability >0.9 were retained.

### The Genetics of Hypertension Associated Treatments (GenHAT) Study

GenHAT [29] is an ancillary study of the Antihypertensive and Lipid-Lowering Treatment to Prevent Heart Attack Trial (ALLHAT). ALLHAT [34] was a randomized, double-blind, multicenter clinical trial with over 42,000 high-risk individuals who had hypertension, aged 55 years or older, and had at least one additional risk factor for cardiovascular disease (CVD). ALLHAT is the largest antihypertensive treatment trial to date and was ethnically diverse, enrolling over 15,000 self-reported Black subjects. Participants were randomized into four groups defined by the class of assigned antihypertensive medication including chlorthalidone, lisinopril, amlodipine, and doxazosin at a ratio of 1.7:1:1:1. Due to a significant increase of major CVD outcomes compared to participants on chlorthalidone, doxazosin was discontinued. The original GenHAT study (*N*=39,114) evaluated the effect of the interaction between candidate hypertensive genetic variants and different antihypertensive treatments on the risk of fatal and non-fatal CVD outcomes. Genotyping was performed using Illumina Infinium Multi-Ethnic AMR/AFR BeadChip (MEGA) arrays on 7546 Black adults who were hypertensive and randomized to either chlorthalidone or lisinopril. Imputation was performed using version r2 of the NHLBI TOPMed reference panel. Participants were excluded if they failed genotyping, had sex mismatch or genotyping call rate <0.95, or if they were an outlier in the PCA (outside of 6 standard deviations). Since ALLHAT used a fasting glucose ≥ 140 mg/dL for the definition of T2D, we excluded controls that had baseline fasting glucose ≥126 mg/dL (*N*=201 excluded) or missing a fasting glucose measure altogether (*N*=1209 excluded). This resulted in 5498 individuals eligible for this study (Table 1). Imputed variants were inspected for their imputation quality scores (*R*^2^) and it was noted that more than 99% of the variants with MAF >1% had an imputation quality >0.6. High-quality genotype calls with genotypic probability >0.9 were retained.

### The Hypertension Genetic Epidemiology Network (HyperGEN)

HyperGEN [30] is a cross-sectional, population-based study and component of the NHLBI Family Blood Pressure Program that was designed to identify genetic risk factors for hypertension and target end-organ damage due to hypertension. The cohort is composed of self-reported White and Black sibships in which at least two siblings were diagnosed with hypertension (defined as either self-reported use of antihypertensive medications or SBP ≥140 mmHg and/or DBP ≥90 mmHg at two separate evaluations) before age 60, their unmedicated adult offspring, and age-matched controls. Later the study population was expanded to include other siblings of the original sibling pair as well as any offspring for a total sample size of *N*=5000. Genotyping on Black participants was performed using whole genome sequencing (WGS), through the NHLBI WGS program (*N*=1896; Table 1). In order to harmonize our imputation efforts with the array-based panels of the REGARDS, GenHAT, and Warfarin (see below) studies, we compiled a set of non-monomorphic and non-multi-allelic SNPs with MAF >1% that were genotyped as part of those studies. This yielded a total of 2,204,415 SNPs that were used as fence post markers for imputing the HyperGEN cohort using version r2 of the NHLBI TOPMed reference panel. Imputed variants were inspected for their imputation quality scores (*R*^2^) and it was noted that more than 99% of the variants with MAF >1% had an imputation quality >0.6. High-quality genotype calls with genotypic probability >0.9 were retained.

### Warfarin Pharmacogenomics Cohort (WPC)

The UAB WPC [31] is a prospective cohort of first-time warfarin users aged 19 years or older starting warfarin for anticoagulation. Warfarin therapy requiring a target international normalized ratio (INR) range of 2-3 was initiated in patients with venous thromboembolism, stroke/transient ischemic attacks, atrial fibrillation, myocardial infarction, and/or peripheral arterial disease. Patients requiring a higher intensity (INR 2.5 to 3.5) or lower intensity (INR 1.5 to 2.5) of anticoagulation were excluded. Baseline demographics, as well as medication history and compliance, were obtained. Changes in INR, medications, and laboratory parameters were documented at each clinical visit as reported previously. Genotyping was performed on the Illumina MEGA array and an Illumina 1M duo array for 599 and 297 self-reported Black participants, respectively. Participants aged 40 years or younger were excluded, leaving 655 individuals (Table 1). Imputation was performed using version r2 of the NHLBI TOPMed reference panel. Imputed variants were inspected for their imputation quality scores (*R*^2^) and it was noted that more than 99% of the variants with MAF >1% had an imputation quality >0.6. High-quality genotype calls with genotypic probability >0.9 were retained.

### The Taiwan Biobank (TWB)

A total of 110,926 TWB participants were genotyped using two different customized arrays (TWBv1: 27,719 samples; TWBv2: 83,207 samples). Due to data release timelines, samples genotyped on the TWBv2 array were divided into two subsets, with 68,975 samples and 14,232 samples, respectively. Quality control (QC) and imputation were performed on the three batches of data separately. Detailed information on the sample characteristics, collection of phenotypes, and QC procedures can be found elsewhere [20, 21].

For each batch, we filtered out variants with genotyping call rate <0.98 and samples with call rate <0.98, and removed variants that were duplicated, monogenic, or not correctly mapped to a genomic position. We then merged TWB samples with 1KG phase 3 data (*N*=2504), and selected high-quality, common variants shared between the two datasets. Next, we performed LD-pruning (PLINK --indep-pairwise 200 100 0.1) and computed PCs of the merged genotype data with LD-pruned variants. Using the population labels of 1KG samples as the reference, we trained a random forest model with top 6 PCs to classify TWB samples into 1KG super-population groups. We retained TWB samples that can be assigned to a homogeneous East Asian group with a predicted probability >0.8 (Additional File 1: Fig. S2). After population assignment, we filtered out outliers in heterozygosity rate and population-specific PCs, and samples with sex mismatch. Imputation was performed using Eagle v2.4 (for pre-phasing) [35] and Minimac4 [36] with 1KG phase 3 data as the reference panel. We randomly removed one sample from each related pair of individuals within or across batches, leaving 25,110, 54,078, and 10,378 individuals for the three batches, respectively (Table 1). Post-imputation QC excluded variants with imputation quality scores <0.6 and MAF <0.5%.

### Evaluation of PRS

We benchmarked the prediction accuracy of the trans-ancestry PRS constructed by PRS-CSx against PRS-CS EUR, PRS-CS Meta, and LDpred2 Meta in the European, African, and Hispanic/Latino samples of the eMERGE I-III genotyped dataset. We additionally assessed the PRS-CSx-derived trans-ancestry PRS in four UAB Black cohorts (i.e., REGARDS, GenHAT, HyperGEN, and WPC) and TWB. For each of these evaluation datasets, we calculated the PRS for each individual by multiplying the number of risk alleles by the algorithm-inferred weights for each variant and summing across the genome using the --score function in PLINK 1.9 [37].

We calculated a range of metrics to assess the predictive performance of the PRS. To measure the overall prediction accuracy, we calculated (i) the proportion of variation in the T2D case-control status explained by the PRS on the liability scale [38], after accounting for a basic set of covariates including age, sex, top 10 PCs of the genetic data, and study site (in the eMERGE analysis only); (ii) the area under the receiver operating characteristic (ROC) curve (AUC) for the covariates-only model (age, sex, top 10 PCs and study site), the PRS-only model, the PRS adjusting for the covariates, and the PRS combined with covariates; (iii) the odds ratio (OR) per standard deviation (OR/SD) change in the PRS, adjusting for the basic covariates. To quantify the discrimination capability at the extreme tail of the PRS, we identified individuals at the top 2%, 5%, or 10% of the PRS distribution, and calculated OR of these high-risk individuals versus the rest of the samples, adjusting for the covariates. We further calculated the sensitivity, specificity, positive predictive value (PPV; the proportion of identified high-risk individuals who are true T2D cases), and negative predictive value (NPV; the proportion of individuals who are not identified as high-risk and are true T2D controls) to examine the clinical utility of these classifiers. Since PPV and NPV depend on the prevalence of the disease, we report prevalence-adjusted PPV and NPV calculated as:

$$\mathrm{PPV}=\left(\mathrm{sensitivity}\times \mathrm{prev}\right)/\left[\mathrm{sensitivity}\times \mathrm{prev}+\left(1-\mathrm{specificity}\right)\times \left(1-\mathrm{prev}\right)\right],$$

$$\mathrm{NPV}=\mathrm{specificity}\times \left(1-\mathrm{prev}\right)/\left[\mathrm{specificity}\times \left(1-\mathrm{prev}\right)+\left(1-\mathrm{sensitivity}\right)\times \mathrm{prev}\right],$$

where prev denotes population-specific prevalence of diagnosed T2D, which was extracted from the recent literature (European 10.0%; African 12.5%; Hispanic 13.1%; Asian 13.7%) [2].

We obtained an overall OR for the African population at various cutoffs by meta-analyzing estimates from the eMERGE African dataset with estimates from the four UAB Black cohorts using an inverse-variance-weighted approach; we obtained an overall OR for the East Asian population at various cutoffs by meta-analyzing the three TWB batches.

### Post hoc ancestry adjustment

To express the trans-ancestry PRS on the same scale across ancestries and facilitate the selection of a single cutoff for clinical implementation of the PRS in prospective cohorts, we evaluated a regression-based ancestry adjustment method that builds on prior work [39, 40] and uses 1KG phase 3 data as the reference panel. Specifically, we calculated the PRS in the full 1KG dataset (*N*=2504), and assumed that both the mean and variance of the PRS depend on the top 5 PCs of the 1KG samples via two linear regressions:

$$\boldsymbol{PRS}\sim {\alpha}_0+{\sum}_{k=1}^5{\alpha}_k\times {\boldsymbol{PC}}_k,\kern2.5em \boldsymbol{\delta} \sim {\beta}_0+{\sum}_{k=1}^5{\beta}_k\times {\boldsymbol{PC}}_k,$$

where ***δ*** is the residual variance of the first regression. Fitting the two regressions gives how individual-level PRS vary with ancestry captured by PCs. For any individual *i* projected into the same PC space with the raw score *PRS*_*i*, raw_ and PC coordinates *PC*_*i*, *k*_, *k* = 1, 2, ⋯, 5, an ancestry adjusted PRS can then be calculated as:

$${PRS}_{i,\mathrm{adj}}=\frac{PRS_{i,\mathrm{raw}}-\left({\hat{\alpha}}_0+{\sum}_{k=1}^5{\hat{\alpha}}_k\times {PC}_{i,k}\right)}{\sqrt{{\hat{\beta}}_0+{\sum}_{k=1}^5{\hat{\beta}}_k\times {PC}_{i,k}}}.$$

We visually examined the PRS distribution in each ancestral group of the eMERGE dataset before and after the adjustment, and evaluated the overall calibration of the adjusted PRS in the prediction model across eMERGE samples by comparing the predicted risk (i.e., the average predicted probability of being a case) and the observed risk (i.e., the proportion of cases) in each PRS decile. Lastly, to assess the impact of the adjustment on the tail discrimination of the trans-ancestry PRS, we compared the OR of individuals in the top percentiles of the adjusted PRS across ancestries by applying a single cutoff with the OR of individuals in the top percentiles of the raw PRS within each ancestry in the multi-ethnic eMERGE dataset.

## Results

### T2D phenotyping in eMERGE

Additional File 2: Table S2 shows the validation results for the three algorithms (eMERGE, modified eMERGE and modified MGB) in the two independent chart review datasets from MGB (138 cases, 70 non-cases; case prevalence 66.3%) and UAB (184 cases, 14 non-cases; case prevalence 92.9%). PPVs ranged from 0.79 to 0.94 with the highest PPV observed for the modified MGB algorithm. Sensitivities ranged from 0.61 to 0.92, with the highest sensitivity observed for the modified eMERGE algorithm. NPVs in the UAB validation dataset were zero for both eMERGE algorithms because all 14 non-cases were classified as cases. Based on these results, we selected the modified MGB algorithm for implementation in eMERGE to define T2D cases and controls for the PRS analysis.

### Evaluation of the PRS in eMERGE

The effect sizes of lead variants in genome-wide significant loci of the T2D meta-GWAS were largely concordant between the European and East Asian populations (correlation of the absolute values of effect sizes across 271 variants: *r* = 0.67), while the effect sizes between the European and African populations were less concordant but still moderately correlated (correlation of the absolute values of effect sizes across 217 variants; *r* = 0.34), suggesting that the cross-ancestry genetic architecture of T2D is consistent with the modeling assumptions in PRS-CSx (Additional File 1: Fig. S3).

Table 1 and Additional File 2: Table S1 show the number of T2D cases and controls in the European, African, and Hispanic/Latino populations, for which we had sufficient sample sizes to evaluate the performance of the PRS, across the 8 eMERGE sites. When the target population was European, the trans-ancestry PRS constructed by PRS-CSx explained 9.2% of the variation in the T2D status on the liability scale, with an AUC of 0.66 and OR/SD of 1.96 (95% confidence interval [CI] 1.91–2.02), after adjusting for age, sex, top 10 genetic PCs and eMERGE study sites (Table 2; Additional File 2: Table S3). The PRS provided predictive power above and beyond the covariates, increasing the AUC from 0.74 (the covariates-only model) to 0.79 when combining covariates and the PRS as predictors (Additional File 2: Table S3). To assess the clinical utility of the trans-ancestry PRS, we compared the risk of T2D among individuals in progressively more extreme cutoffs of the PRS distribution relative to the rest of the samples. Individuals of European ancestry in the top decile of the trans-ancestry PRS had an OR of 3.19 (95% CI: 2.97–3.42; *P* value = 1.33E−232) compared with individuals in the bottom 90% of the PRS distribution. Risk continued to increase when contrasting the top 5% of the PRS (OR = 3.55, 95% CI: 3.24–3.90; *P* value = 1.88E−158) and the top 2% of the PRS (OR = 4.21, 95% CI: 3.66–4.84; *P* value = 1.82E−89) to the lower tails of the PRS distribution (Table 2; Additional File 2: Table S3). This shows that the trans-ancestry PRS can identify individuals of European ancestry with a significantly increased risk of T2D. Using the top 2% of the PRS as the classifier, the prevalence-adjusted PPV and NPV were 0.26 and 0.90, respectively (Table 2; Additional File 2: Table S3).

**Table 2:** Prediction accuracy of the trans-ancestry T2D PRS in eMERGE across three populations

Population	Liability *R*^2^	Covariates-adjusted AUC	OR per SD (95% CI)	Top 2% PRS					
				OR (95% CI)	*P* value	Sensitivity	Specificity	Adjusted PPV*	Adjusted NPV*
European	9.2%	0.66	1.96 (1.91, 2.02)	4.21 (3.66, 4.84)	1.82E−89	0.05	0.99	0.26	0.90
African	2.8%	0.58	1.54 (1.46, 1.64)	1.98 (1.43, 2.74)	4.34E−05	0.03	0.98	0.21	0.88
Hispanic	8.0%	0.63	2.08 (1.84, 2.35)	6.87 (3.11, 15.15)	1.81E−06	0.04	0.99	0.43	0.87

*Adjusted PPV and NPV are calculated using the following population-specific prevalence: European 10%; African 12.5%; Hispanic 13.1%

When using the trans-ancestry PRS as a predictor of T2D status for eMERGE individuals of African ancestry, the variance explained on the liability scale was 2.8%, the covariates-adjusted AUC was 0.58, and the OR/SD was 1.54 (95% CI: 1.46–1.64; Table 2; Additional File 2: Table S3). As expected, the prediction accuracy of the PRS in the African population was substantially lower compared with the prediction in the European population, reflecting the current Eurocentric bias in genomic studies and the fact that our trans-ancestry PRS was constructed from GWAS of predominantly European and East Asian individuals. Nevertheless, the T2D risk increased with higher PRS values, and the OR comparing individuals in the top 2% of the PRS distribution with the rest of the individuals was 1.98 (95% CI: 1.43–2.74; *P* value = 4.34E−05), indicating that the trans-ancestry PRS can be useful in identifying individuals with elevated T2D risk in the African population (Table 2; Additional File 2: Table S3).

The overall prediction accuracy of the trans-ancestry PRS in the Hispanic/Latino group was between that observed in the European and African individuals: variance explained = 8.0%; covariates-adjusted AUC = 0.63; OR/SD = 2.08 (Table 2; Additional File 2: Table S3), reflecting that the Hispanic/Latino population is a recent admixture among Europeans, Africans, and Native Americans. Hispanic individuals with high PRS had a substantially increased risk of T2D, with an OR of 6.87 (95% CI: 3.11–15.15; *P* value = 1.81E−06) when contrasting the top 2% of the PRS with the remaining 98% of the distribution (Table 2; Additional File 2: Table S3). However, we note that due to the relatively small sample size of the Hispanic/Latino sample in the eMERGE study, the OR estimates for the tails of the PRS distribution were associated with large uncertainties.

### Comparison with alternative PRS in eMERGE

**Fig. 2:**
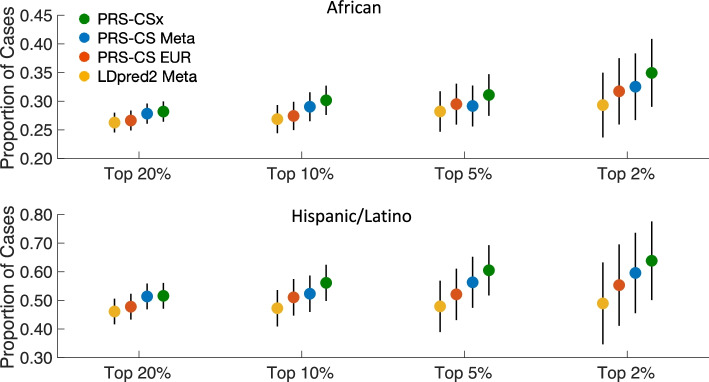
Comparison of the predictive performance of PRS-CSx with three alternative PRS construction methods in the African and Hispanic/Latino samples of the eMERGE dataset. Alternative PRS methods include (i) a European-specific score derived by applying PRS-CS-auto to the European T2D GWAS summary statistics (PRS-CS EUR); (ii) a trans-ancestry score derived by applying PRS-CS-auto to the meta-analysis of the European, MEDIA and BBJ T2D GWAS (PRS-CS Meta); and (iii) a trans-ancestry score derived by applying LDpred2-auto to the T2D meta-GWAS (LDpred2 Meta)

We compared the trans-ancestry PRS constructed by PRS-CSx with a European-specific score derived by applying PRS-CS-auto to the European T2D GWAS summary statistics (PRS-CS EUR), and two alternative trans-ancestry scores derived by applying PRS-CS-auto and LDpred2-auto to the T2D meta-GWAS, denoted as PRS-CS Meta and LDpred2 Meta, respectively. PRS-CSx showed better overall prediction accuracy and tail discrimination (Additional File 2: Table S3) and identified more cases in the top percentiles of the PRS distribution than alternative methods in the African and Hispanic/Latino samples of the eMERGE dataset (Fig. 2). The improvement of PRS-CSx relative to PRS-CS EUR highlights the importance of integrating GWAS from diverse ancestries to increase the portability of PRS to non-European populations, while the improvement of PRS-CSx relative to PRS-CS Meta and LDpred2 Meta demonstrates the benefits of explicitly modeling population-specific allele frequencies and LD patterns.

### Evaluation of the PRS in UAB Black cohorts

Given that the prediction accuracy of the trans-ancestry PRS in the eMERGE African samples was relatively low compared with other populations, we performed further evaluation of the PRS-CSx-derived PRS in four UAB Black cohorts, namely REGARDS (1659 T2D cases, 5086 T2D controls), GenHAT (2776 cases, 2722 controls), HyperGEN (402 cases, 1494 controls) and WPC (300 cases, 355 controls) (Table 1). REGARDS, GenHAT and HyperGEN had largely consistent estimates of performance metrics, and the prediction accuracy was higher relative to the prediction in the eMERGE African samples. Specifically, the AUC of the PRS, adjusting for age, sex, and top 10 genetic PCs, was approximately 0.61, and the OR/SD estimates ranged from 1.70 to 1.85 across the three cohorts (Table 3; Additional File 2: Table S4). OR estimates contrasting individuals in the tail of the PRS distribution with the rest of the samples increased when more extreme cutoffs were applied; at the top 2% of the PRS distribution, an approximately three-fold increase in T2D risk was observed relative to the remaining 98% of the individuals (REGARDS: OR = 3.04, *P* value = 3.87E−10; GenHAT: OR = 2.70, *P* value = 8.44E−06; HyperGen: OR = 3.37, *P* value = 5.37E−04). The prevalence-adjusted PPVs ranged from 0.26 to 0.34 and all prevalence-adjusted NPVs were around 0.88. WPC had the lowest sample size among the four cohorts and had limited power to assess the tail discrimination of the trans-ancestry PRS, but the OR estimate at the top 2% of the PRS was comparable to the other three cohorts, though not statistically significant (OR = 2.7, 95% CI: 0.80–9.09, *P* value = 1.09E−01; Table 3; Additional File 2: Table S4).

**Table 3:** Prediction accuracy of the trans-ancestry T2D PRS in four Black cohorts

Cohort	Liability *R*^2^	Covariates-adjusted AUC	OR per SD (95% CI)	Top 2% PRS					
				OR (95% CI)	*P* value	Sensitivity	Specificity	Adjusted PPV*	Adjusted NPV*
REGARDS	4.6%	0.61	1.70 (1.58, 1.84)	3.04 (2.15, 4.31)	3.87E−10	0.04	0.99	0.30	0.88
GenHAT	3.6%	0.61	1.85 (1.70, 2.01)	2.70 (1.74, 4.18)	8.44E−06	0.03	0.99	0.26	0.88
HyperGen	6.2%	0.62	1.75 (1.52, 2.02)	3.37 (1.69, 6.69)	5.37E−04	0.05	0.99	0.34	0.88
Warfarin	1.5%	0.57	1.37 (1.13, 1.65)	2.70 (0.80, 9.09)	1.09E−01	0.01	0.98	0.07	0.87

*Adjusted PPV and NPV are calculated using 12.5% for the African population

### Evaluation of the PRS in TWB

Since the low sample size of Asian individuals in the eMERGE study precluded PRS analysis, we sought to evaluate our PRS-CSx-derived trans-ancestry PRS in the Taiwan Biobank (TWB), in which participants were predominantly Han Chinese. Analysis in the three batches of TWB data (batch 1: 1248 cases, 23,862 controls; batch 2: 2806 cases, 51,272 controls; batch 3: 516 cases, 9862 controls; Table 1) produced highly consistent results. The trans-ancestry PRS was strongly associated with self-reported T2D status, with the variance explained on the liability scale ranged from 12.9 to 15.3%, the covariates-adjusted AUC ranged from 0.68 to 0.70, and the OR/SD ranged from 2.01 to 2.19 (Table 4; Additional File 2: Table S5). The tail of the PRS was highly discriminative; individuals in the top decile of the PRS had a more than three-fold increase in T2D risk relative to those outside the top decile, and the OR increased to approximately 4.50 when using top 2% of the PRS to identify high-risk individuals (batch 1: OR = 4.62, *P* value = 7.47E−31; batch 2: OR = 4.60, *P* value = 1.80E−69; batch 3: OR = 4.35, *P* value = 1.43E−12). The corresponding prevalence-adjusted PPVs ranged from 0.36 to 0.38, and the prevalence-adjusted NPVs were 0.87 across the three batches (Table 4; Additional File 2: Table S5). Overall, the trans-ancestry PRS was slightly more predictive in this East Asian sample compared with the eMERGE European samples, likely reflecting the contributions from the large BBJ T2D GWAS in the training dataset, the more homogeneous community-based TWB samples relative to the eMERGE study in which sample characteristics may vary across different health care systems, and differences in T2D case-control definitions (self-report vs. EMR-based phenotyping).

**Table 4:** Prediction accuracy of the trans-ancestry T2D PRS in the Taiwan Biobank (TWB)

Batch	Liability *R*^2^	Covariates-adjusted AUC	OR per SD (95% CI)	Top 2% PRS					
				OR (95% CI)	*P* value	Sensitivity	Specificity	Adjusted PPV*	Adjusted NPV*
Batch 1	15.1%	0.70	2.19 (2.05, 2.33)	4.62 (3.56, 5.99)	7.47E−31	0.07	0.98	0.37	0.87
Batch 2	12.9%	0.68	2.01 (1.93, 2.10)	4.60 (3.88, 5.45)	1.80E−69	0.07	0.98	0.38	0.87
Batch 3	15.3%	0.70	2.16 (1.96, 2.38)	4.35 (2.89, 6.53)	1.43E−12	0.06	0.98	0.36	0.87

*Adjusted PPV and NPV are calculated using 13.7% for the Asian population

### Meta-analysis

**Fig. 3:**
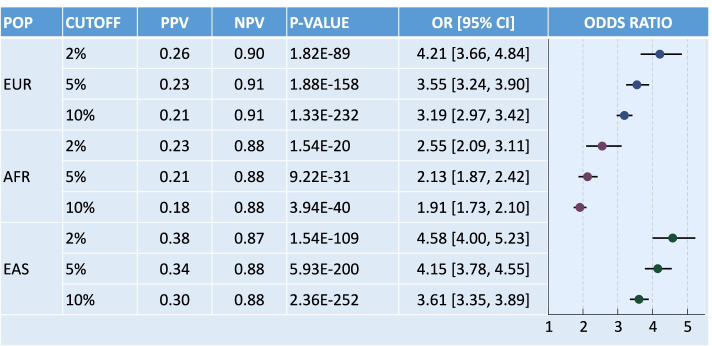
Tail discrimination of the PRS-CSx-derived trans-ancestry T2D PRS at various percentage cutoffs in the European, African (by meta-analyzing the eMERGE African dataset with the four UAB Black cohorts) and East Asian (by meta-analyzing the three TWB batches) populations. POP, population; EUR, European; AFR, African; EAS, East Asian; PPV, prevalence-adjusted positive predictive value; NPV, prevalence-adjusted negative predictive value

To summarize the performance of the trans-ancestry PRS across cohorts, we derived overall OR, and prevalence-adjusted PPV and NPV estimates at various percentage cutoffs for the African population by meta-analyzing the eMERGE African dataset with the four UAB Black cohorts, and overall performance metrics for the East Asian population by meta-analyzing the three TWB batches (Additional File 2: Table S6). Along with the eMERGE European dataset, at least 4500 T2D cases were available for the three populations, enabling an accurate assessment of the tail discrimination of the trans-ancestry PRS. We note that while there was a substantial difference in case prevalence across eMERGE African and UAB Black cohorts, the performance metrics of the trans-ancestry PRS (e.g., AUC, PPV, and NPV) appeared to be robust to this variation. Figure 3 shows that the PRS was highly significantly associated with T2D status across different percentage cutoffs and ancestral groups, with comparable predictive performance in the European and East Asian populations and lower prediction accuracy in the African population. Individuals in the top 2% of the PRS distribution had significantly increased T2D risk, with the OR estimates ranging from 2.55 in the African samples to 4.58 in the East Asian samples, which corresponds to the increased risk of T2D for first-degree relatives [41] and suggests a clinical value of the trans-ancestry PRS in diverse populations.

### Ancestry adjustment

While the trans-ancestry PRS was significantly associated with T2D status across the ancestral groups we examined and showed potential for clinical translation, the raw PRS had major distributional shifts by ancestry (Additional File 1: Fig. S4; left panel), impeding its implementation in clinical care where a single cutoff of the PRS distribution across diverse populations is needed to identify high-risk individuals. By modeling the mean and variance of the trans-ancestry PRS as functions of population structure captured by genetic PCs, the post hoc ancestry adjustment method eliminated major distributional differences in the PRS across ancestries in the eMERGE dataset (Additional File 1: Fig. S4; right panel). The adjusted PRS showed good overall calibration across eMERGE samples, as demonstrated by largely concordant predicted and observed risk (i.e., the proportion of cases) in each PRS decile (Additional File 1: Fig. S5). To examine the impact of this post hoc adjustment on the tail discrimination of the PRS, we compared the OR of individuals in the top percentiles of the adjusted PRS across ancestries with the OR of individuals in the top percentiles of the raw PRS within each ancestry (i.e., OR estimates reported in Table 2 and Additional File 2: Table S3). Additional File 2: Table S7 shows that other than the OR estimate at top 2% of the PRS in the Hispanic/Latino samples, which was affected by a relatively low sample size and large estimation uncertainties, high-risk individuals identified by the adjusted PRS using a single cutoff showed comparable or slightly higher OR estimates than those identified by the raw PRS separately in each ancestral group, suggesting that the ancestry adjustment method did not compromise the predictive performance of the trans-ancestry PRS.

## Discussion

We have shown that the top 2% of a trans-ancestry PRS distribution can identify individuals of European, African, Hispanic/Latino, and East Asian ancestry with a roughly 2.5–4.5-fold of increase in T2D risk, which corresponds to numerous studies that showed a similar increased risk of T2D for first degree relatives (see e.g., [41]). By integrating GWAS summary statistics from multiple populations using PRS-CSx, the trans-ancestry PRS was significantly associated with T2D status in all populations examined, providing a robust and potentially clinically meaningful index of risk among diverse patients in clinical settings.

Recent studies have demonstrated in European individuals that T2D PRS can provide predictive power for incident T2D above and beyond established risk factors such as age, body mass index (BMI), smoking, physical activity levels, and history of high glucose and hypertension and can identify high-risk individuals and stratify lifetime risk trajectories of T2D patients [42, 43], suggesting potential for clinical translation. However, most existing T2D scores were developed and validated in individuals of European descent. As the interest in the clinical implementation of PRS for common diseases like T2D continues to grow, a major challenge is the uncertainty about how best to combine multi-ethnic GWAS and estimate polygenic risk in diverse populations.

In research settings, trans-ancestry PRS are often derived from multi-ethnic meta-GWAS [7, 44] or trained in each target population separately [45, 46]. However, the former approach does not model population-specific allele frequency and LD patterns, which limits the performance of PRS, while the latter approach requires assigning individuals to discrete ancestral groups to optimize PRS estimation, which is challenging in clinical applications because self-reported race/ethnicity may be inconsistent with genetic ancestry. The patient group for returning PRS results may also contain admixed individuals who cannot fit into ancestry categories easily. Communication of PRS results in clinical settings would thus be facilitated by development of a trans-ancestry PRS without stratifying patients into individual ancestral groups.

In this work, we used a fully Bayesian polygenic modeling method, PRS-CSx, to derive SNP weights for a trans-ancestry PRS without the need of a priori population assignment or hyper-parameter tuning. PRS-CSx jointly models GWAS summary statistics across populations and explicitly accounts for population-specific allele frequencies and LD patterns. While non-European GWAS are often less powerful than European studies, they inform the genetic architecture in non-European populations and may capture population-specific genetic risk factors. Integrating available GWAS across ancestral groups may thus improve the portability of PRS -- especially to non-European populations -- and deliver personalized risk prediction that can more equally benefit all populations. Compared with early T2D PRS developed using a small number of SNPs selected based on statistical and/or biological significance (see [47] for a review), more recent T2D PRS derived from large-scale European GWAS [8, 9, 48] or multi-ethnic meta-GWAS [7, 44], and PRS evaluated in this work using more sophisticated PRS construction methods such as PRS-CS and LDpred2, our trans-ancestry PRS constructed by PRS-CSx demonstrated improved prediction accuracy and transferability across ancestral groups, reflecting the combined effect of methodological advances and increased sample sizes in the training GWAS. We note that the T2D GWAS of African ancestry from the MEDIA Consortium was substantially underpowered relative to the GWAS in European and East Asian populations, which limited the performance of PRS-CSx. As the diversity and scale of discovery GWAS continue to expand, principled computational models that can appropriately integrate multi-ancestry genome-wide data are expected to further improve the prediction in non-European populations and demonstrate bigger advantages over single-population methods. In addition, while HapMap3 variants represent a good balance between computational cost and prediction accuracy for PRS construction in European populations, they may tag a lower proportion of genetic variation in non-European populations, limiting the transferability of PRS. Future work is needed to develop computationally efficient algorithms that can incorporate genome-wide genetic variants into PRS calculation.

While the trans-ancestry PRS derived by PRS-CSx showed promise for clinical translation when evaluated separately in each population, implementation of the PRS in a prospective cohort with individuals from diverse ancestry backgrounds requires calibrated PRS distributions across populations. In this work, we evaluated a post hoc ancestry adjustment method that can express the polygenic risk on the same scale across ancestries without compromising the discrimination capability at the extreme tails of the PRS. With this adjustment, a single cutoff of the PRS distribution for the high-risk group can be identified and applied to any target individual. We expect that using large and diverse reference panels that better match the population structure of the prospective cohort will facilitate more accurate ancestry adjustment and risk estimation.

Our study has several limitations: (i) the gap in the prediction accuracy between European/Asian and African populations remains considerable, likely due to the underrepresentation of African samples in the training GWAS; (ii) evaluation samples other than Europeans, Africans and East Asians were limited; (iii) the selection of high-risk individuals at extreme tails of the PRS distribution may be associated with uncertainties [49], which has not been fully characterized in this work; (iv) the sample characteristics of the evaluation cohorts may not fully represent the patient group to which PRS results will be returned, and the effects of PRS may depend on ascertainment and vary across different target samples; (v) predictive performance of the PRS was only assessed for prevalent T2D cases; and (vi) the associations between the trans-ancestry PRS and standard clinical risk factors (e.g., BMI, low physical activity, and hypertension), and the value of the PRS over and above these factors remain unclear. Evaluating the capability of the trans-ancestry PRS in identifying incident cases (i.e., individuals at risk to develop the disease in a future time window) in prospective cohorts and combining common-variant PRS with other genetic and non-genetic risk factors and family history into an integrated risk estimate would better define the clinical impact of the PRS.

Previous studies on returning PRS to patients have produced mixed results. For example, the Genetic Counseling/Lifestyle Change (GC/LC) study, which delivered a genetic score constructed from 36 T2D-associated SNPs to overweight patients at risk for T2D, reported limited changes in lifestyles and prevention program adherence compared with controlled participants who did not receive genetic counseling [50–52]. The MedSeq Project, which returned polygenic risk estimates for cardiometabolic traits along with monogenic disease risk, pharmacogenomic associations, and family history to patients and their healthcare providers (HCPs), found that genetic testing may prompt additional clinical actions of unclear value [53]. In contrast, the MI-GENES study provided evidence that disclosure of the genetic risk of coronary heart disease led to greater statin use and lower low-density lipoprotein cholesterol levels after 6 months than returning conventional risk factors alone [54]. The different conclusions drawn from these clinical trials may be partly explained by the many factors that influence the effectiveness of returning PRS to patients, including the understanding of the implications of polygenic risk by patients and HCPs, the change of healthcare services based on personalized risk estimates (e.g., surveillance of patients, ordering screening tests, prescribing preventive medications, or providing lifestyle advice), communication of risk to patients, the availability of risk management protocols, and uptake of and adherence to risk-reduction recommendations. Expanded research on the best practice of returning genetic testing results in clinical settings will clarify the benefits of adding polygenic risk estimates to clinical risk factors and family history to create an integrated risk assessment.

## Conclusions

In summary, we have constructed and evaluated a trans-ancestry T2D PRS in multiple populations. Future work is needed to expand the scale of non-European genomic resources and conduct larger GWAS in underrepresented populations to further increase the transferability of PRS, assess the predictive performance of the PRS in more diverse samples and real-world settings, refine risk communications, and monitor the impact of returning risk estimates on related clinical outcomes, in order to ensure equitable and effective deployment of PRS to clinical care.

## Supplementary Information

**Additional File 1: Supplementary Figures S1-S5.**

**Additional File 2: Supplementary Tables S1-S7.**

## Data Availability

GWAS summary statistics from the Mahajan et al. 2018 study [8] are publicly available at the DIAGRAM consortium website (http://diagram-consortium.org). Biobank Japan (BBJ) T2D GWAS summary statistics [23] are publicly available on the BBJ website (https://pheweb.jp). GWAS summary statistics from the MEDIA study [22] are available on the database of Genotypes and Phenotypes (dbGaP; Study Accession: phs000930.v9.p1). The eMERGE phenotypic and genetic data are available on dbGaP (Study Accession: phs001584.v2.p2). Individual-level phenotypic and genetic data of REGARDS (Study Accession: phs002719.v1.p1) and GenHAT (Study Accession: phs002716.v1.p1) are in the process of being deposited in dbGaP. While awaiting data release via dbGaP, investigators may contact Dr. Marguerite R. Irvin (irvinr@uab.edu) to discuss gaining access to or collaborating on the REGARDS and GenHAT data. Individual-level data from the HyperGEN study are available on dbGaP (Study Accession: phs001293.v3.p1). Researchers interested in obtaining TWB individual-level data for research purposes would need to submit an application that includes a detailed research proposal and an Institutional Review Board (IRB) approval to TWB (biobank@gate.sinica.edu.tw). The application will undergo scientific and ethical reviews by external experts in relevant scientific fields and the TWB ethical governance committee (EGC). Researchers will be able to access TWB data for approved research projects during the approved time period. For international researchers outside of Taiwan, an additional international data transfer agreement needs to be filed to the Ministry of Health and Welfare of Taiwan to enable sharing of TWB individual-level data and any derived data. Posterior SNP effect size estimates for the trans-ancestry T2D PRS generated by PRS-CSx are available at https://github.com/getian107/PRScsx, and are deposited in the PGS Catalog (https://www.pgscatalog.org; PGS ID: PGS002308).

## Abbreviations

T2D: Type 2 diabetes (T2D)
GWAS: Genome-wide association studies
PRS: Polygenic risk scores
eMERGE: Electronic Medical Records and Genomics
MGB: Mass General Brigham
EMR: Electronic medical records
MEDIA: MEta-analysis of type 2 Diabetes in African Americans
BBJ: Biobank Japan
TWB: Taiwan Biobank
REGARDS: Reasons for Geographic and Racial Differences in Stroke Study
GenHAT: The Genetics of Hypertension Associated Treatments Study
HyperGEN: The Hypertension Genetic Epidemiology Network
WPC: Warfarin Pharmacogenomics Cohort
OR: Odds ratio
PPV: Positive predictive value
NPV: Negative predictive value
AUC: Area under the receiver operating characteristic curve
PC: Principal component
HCP: Healthcare providers
