# Supplementary material for: Development and validation of a trans-ancestry polygenic risk score for type 2 diabetes in diverse populations
Source: Genome Med. 2022 Jun 29;14:70. doi: 10.1186/s13073-022-01074-2 (PMC9241245; doi:10.1186/s13073-022-01074-2)
Supplement: Supplementary file 1 — Additional File 1: Supplementary Figures S1-S5. [file 13073_2022_1074_MOESM1_ESM.pdf]

Supplementary Figures for *Development and Validation of a Trans-Ancestry Polygenic Risk Score for Type 2 Diabetes in Diverse Populations*

Tian Ge, Marguerite R. Irvin, Amit Patki, Vinodh Srinivasasainagendra, Yen-Feng Lin, Hemant K. Tiwari, Nicole D. Armstrong, Barbara Benoit, Chia-Yen Chen, Karmel W. Choi, James J. Cimino, Brittney H. Davis, Ozan Dikilitas, Bethany Etheridge, Yen-Chen Anne Feng, Vivian Gainer, Hailiang Huang, Gail P. Jarvik, Christopher Kachulis, Eimear E. Kenny, Atlas Khan, Krzysztof Kiryluk, Leah Kottyan, Iftikhar J. Kullo, Christoph Lange, Niall Lennon, Aaron Leong, Edyta Malolepsza, Ayme D. Miles, Shawn Murphy, Bahram Namjou, Renuka Narayan, Mark J. O'Connor, Jennifer A. Pacheco, Emma Perez, Laura J. Rasmussen-Torvik, Elisabeth A. Rosenthal, Daniel Schaid, Maria Stamou, Miriam S. Udler, Wei-Qi Wei, Scott T. Weiss, Maggie C. Y. Ng, Jordan W. Smoller, Matthew S. Lebo, James B. Meigs, Nita A. Limdi, Elizabeth W. Karlson

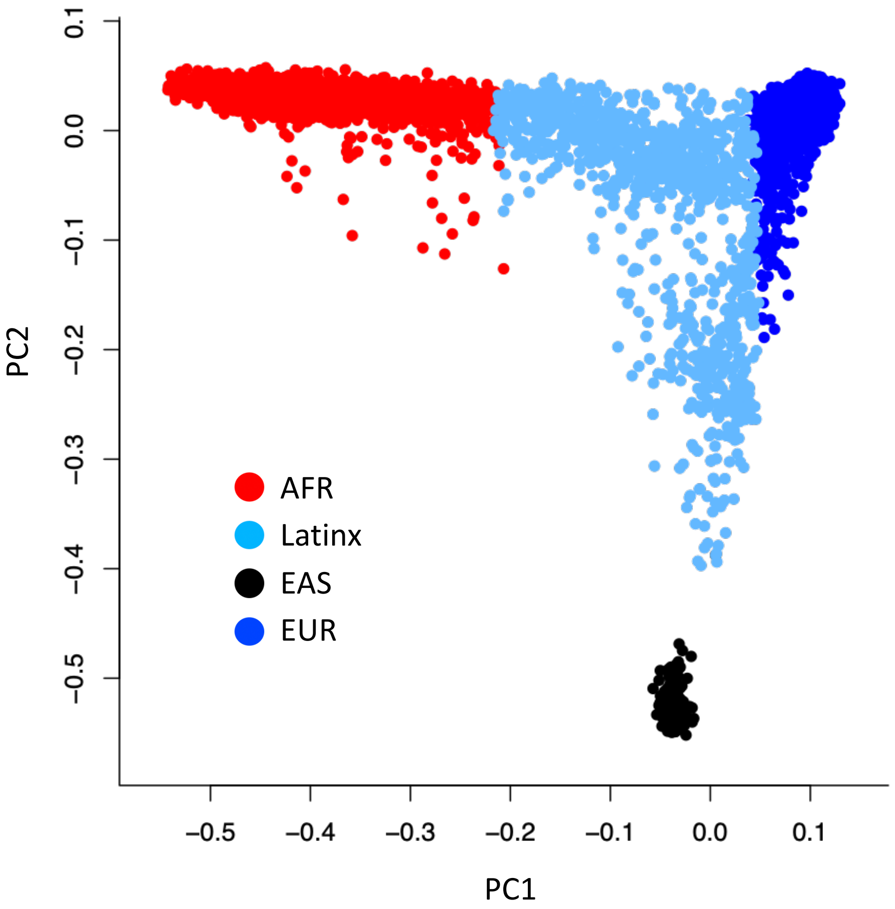

Figure S1: The first two genetic principal components of the final analytic samples of the eMERGE dataset. AFR = African; Latinx = Hispanic/Latino; EAS = East Asian; EUR = European.

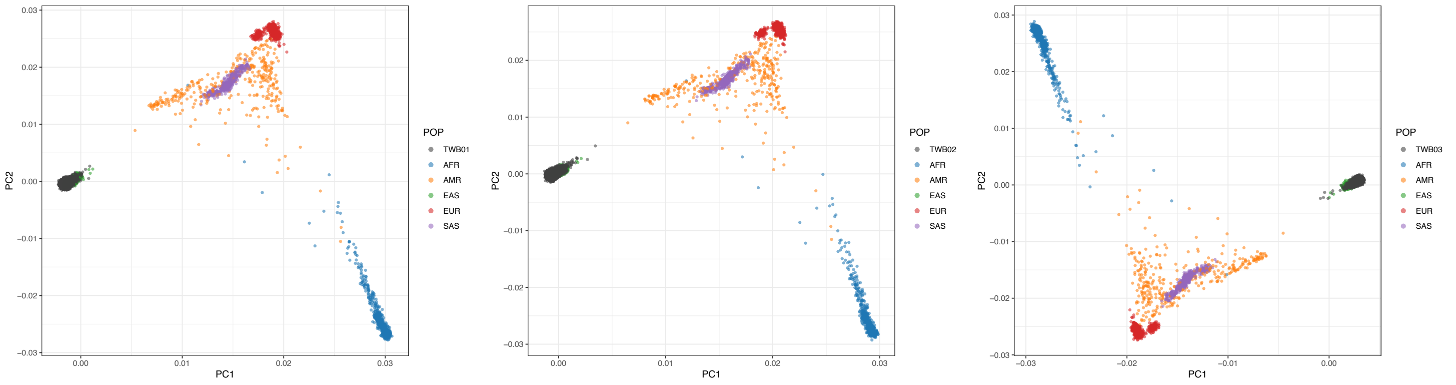

Figure S2: The first two genetic principal components of the final analytic samples of the Taiwan Biobank, with the 1000 Genomes Project Phase 3 samples overlaid. POP = Population; AFR = African; AMR = Admixed American; EAS = East Asian; EUR = European; SAS = South Asian.

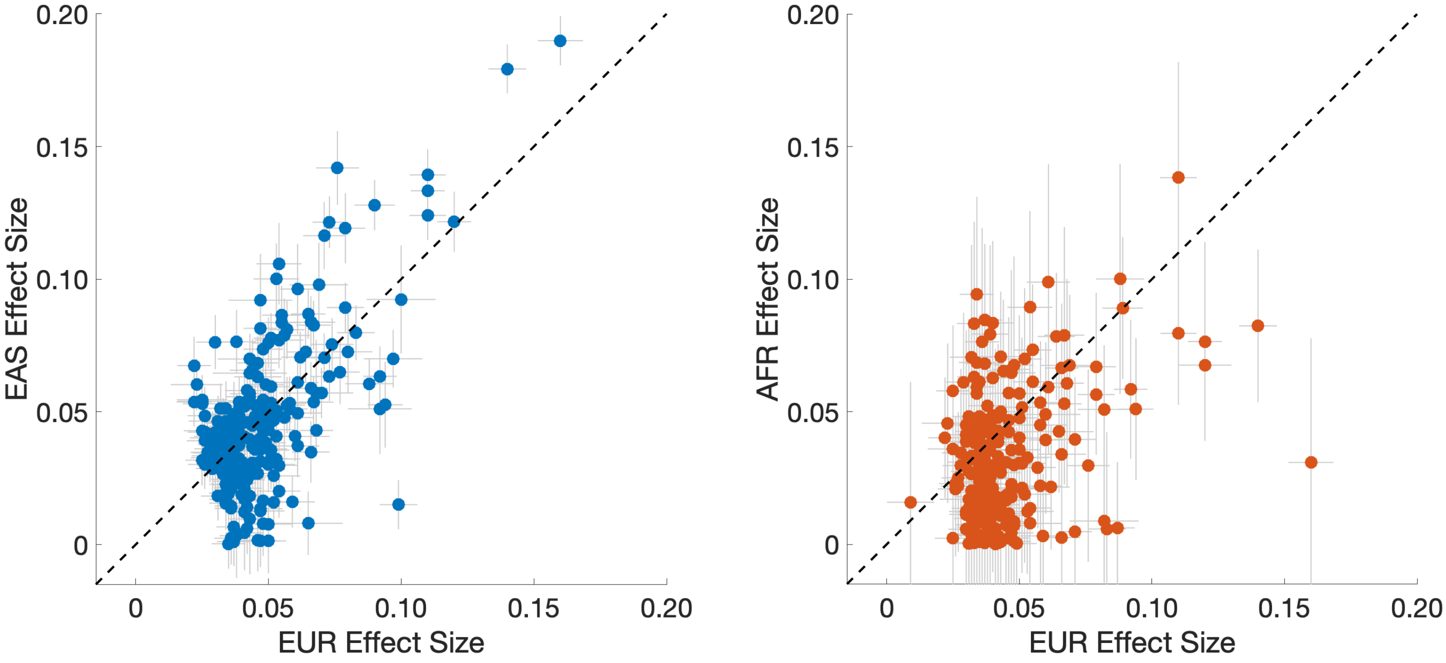

Figure S3: Concordance of the genetic effects on T2D across populations. Each dot represents the lead variant (or, in the case where the lead variant is missing in a population, a tag variant that is strongly correlated with the lead variant) of a genome-wide significant locus in the meta-analysis of the European, African and East Asian T2D GWAS. Error bars represent 95% confidence intervals.

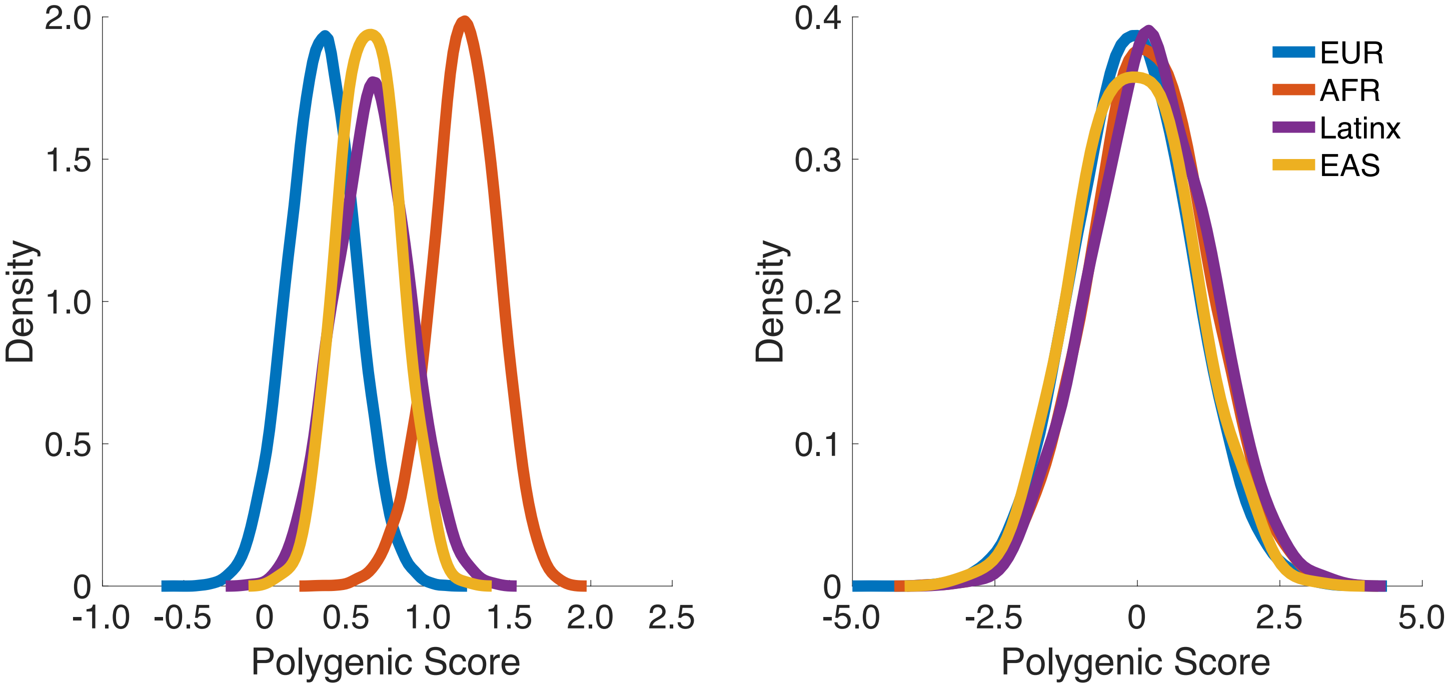

Figure S4: The distribution of the PRS-CSx-derived trans-ancestry PRS by population before (left panel) and after (right panel) the post-hoc ancestry adjustment. EUR = European; AFR = African; Latinx = Hispanic/Latino; EAS = East Asian.

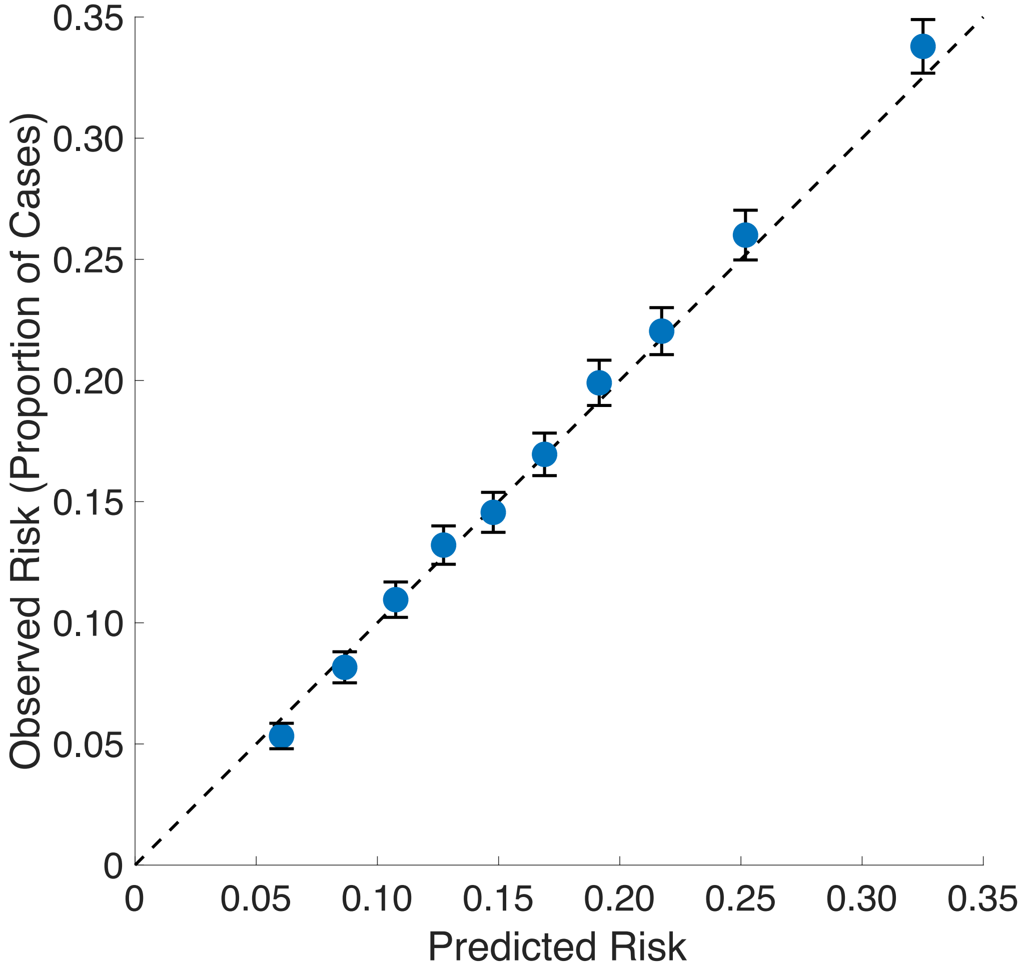

Figure S5: Calibration of the prediction of the PRS-CSx-derived trans-ancestry PRS in the full eMERGE samples. The figure shows the observed risk (i.e., the proportion of cases) against the predicted risk (i.e., the average predicted probability of being a case) in each decile of the PRS. Error bars represent 95% confidence intervals.
